# Implementation experience of a 12-month intervention to introduce intermittent kangaroo mother care to eight Chinese neonatal intensive care units

**DOI:** 10.1007/s12519-022-00607-4

**Published:** 2022-08-25

**Authors:** Xin Liu, Xiao-Hui Chen, Zhan-Kui Li, Bei Cao, Shao-Jie Yue, Qiong-Yu Liu, Chuan-Zhong Yang, Chang-Yi Yang, Ying-Xi Zhao, Geng-Li Zhao, Qi Feng

**Affiliations:** 1 Neonatal Intensive Care Unit, Department of Pediatrics, Peking University First Hospital, Beijing 100034, China; 2 Department of Neonatology, Nanjing Maternity and Child Health Care Hospital, Nanjing, China; 3 Department of Neonatology, Northwest Women’s and Children’s Hospital, Xi’an, China; 4 Department of Neonatology, The Maternal and Child Health Care Hospital of Hunan Province, Changsha, China; 5 Department of Neonatology, Xiangya Hospital Central South University, Changsha, China; 6 Department of Neonatology, Women & Children’s Health Care Hospital of Linyi, Linyi, China; 7 Department of Neonatology, Shenzhen Maternity & Child Healthcare Hospital, Shenzhen, China; 8 Department of Neonatology, Fujian Provincial Maternity and Children’s Hospital, Fuzhou, China; 9 Nuffield Department of Medicine, University of Oxford, Oxford, UK; 10 Department of Obstetrics and Gynecology, Peking University First Hospital, Beijing, China

Kangaroo mother care (KMC) is recommended by the World Health Organization for the care of preterm and low-birth-weight newborns. KMC has been shown to increase survival rates and the quality of life of preterm and low-birth-weight infants, including improved clinical outcomes, weight gain and thermoregulation when compared to conventional care [1–3]. More recent research focused on KMC in high-risk preterm, critically ill newborns and newborns requiring special care, which suggested that KMC helped to stabilize the vital signs of preterm infants [4, 5]. KMC is well accepted and promoted by medical associations and professional organizations around the world [6, 7]. In November 2015 with support from the National Health Commission of China, participants from ten hospitals based in different areas of China received theoretical and practical training on KMC. During a four-year period, health professionals from these hospitals participated in theoretical and practical training, which included two operation training from foreign experts, two academic lectures, and three study tours to hospitals in the UK, Sweden and the Netherlands. During this period, the KMC Operational Manual for Premature Infants in China was developed, and a 12-month prospective multicenter study of KMC for preterm infants was conducted in the eight hospitals. In the present study, we further examine whether KMC was sustained as a practice during a 12-month period from April 2018 to March 2019 and the characteristics of preterm infants that received KMC.

This multicenter study was conducted in eight neonatal intensive care units (NICUs) across China from April 1, 2018, to March 31, 2019. Our study population was preterm infants [gestational age (GA) < 37 weeks] that received KMC during their hospital stay in the NICU. KMC provision followed the standard clinical protocol and the flowchart developed by the study team. An infant’s eligibility for KMC was determined by local neonatologists based on their perceived benefits of KMC, babies’ conditions and service capacity of each NICU. Nursing staff was available for help at any time.

One designated member of staff from each NICU was in charge of data collection, entry and verification. All data then were compiled, reviewed and analyzed by the authors in a central location (Peking University First Hospital). Data analysis was conducted using SPSS for Windows version 20 (IBM Inc. Chicago, IL). The test level was set at α = 0.05, and *P* < 0.05 was considered statistically significant. Statistical significance was determined by Chi-square test, Student’s *t* test or by nonparametric rank sum test based on the outcome of interest. Also, linear mixed regression models assessed the association between weight change and different measures, time effects and predictors were included as fixed effects, the weight of inter-individual and inter-center variations were excluded to fulfill the independence assumptions of linear models. The model-based approach was extended to analyze the multiple groups of infants by adding dummy variables for additional main effects and their interactions with time. The patient and center analyses were performed as random effect analyses, and the significance level was defined as 0.05.

A total of 8240 infants were enrolled in the study, and 2093 (25.4%) received KMC (Supplementary Table 1). The percentage of preterm infants that received KMC remained relatively stable during the study period between 23.8% and 27.7%. The majority of infants (31.4%) had a birth weight of between 2000 and 2500 g, 20.2% of infants had a birth weight of less than 1500 g. Preterm infants with a GA of less than 32 weeks were most likely to receive KMC during their stay in NICU (59.8% ± 5.6%). On average there were more infants who received KMC with a GA at birth between 28 and 32 weeks (49.6% ± 4.7%) than infants in other GA categories, and this group had the largest proportion across 12 months (Supplementary Fig. 1). The majority of infants who received KMC had a birth weight of 1000–1500 g (41.9% ± 2.9%) and 1500–2000 g (36.4% ± 4.5%), respectively (Supplementary Fig. 2). The average GA at birth, length of hospital stay and corrected gestational age (CGA) at discharge were similar across the 12-month study period (Table 1). The neonatal respiratory distress syndrome occupied a large proportion, ranging from 27.2% to 34.4%. On average infants received their first session of KMC at 18–21 days of age, 33–34 weeks CGA and 1038.2–2263.2 g body weight, and one-third of these infants required respiratory support when KMC was initiated. The accumulated length of time of KMC provided to each infant increased from an average of 12 to 14 hours.

**Table 1:** Average characteristics of infants who received kangaroo mother care with a birth weight < 2000 g

Items	Q1: Apr-18 to Jun-18	Q2: Jul-18 to Sep-18	Q3: Oct-18 to Dec-18	Q4: Jan-19 to Mar-19	*χ*^2^/*F*	*P*
Gestational age at birth (wk)	30.6 ± 2.3	30.6 ± 2.4	30.5 ± 2.5	30.6 ± 2.3	0.3	0.9
Birth weight (g)	1388.3 ± 294.5	1381.1 ± 310.0	1367.4 ± 320.5	1413.3 ± 320.8	1.4	0.2
Length of hospital stay (d)	41.7 (26, 51)	43.9 (28, 52)	40.0 (27, 56)	39.0 (27, 55)	2.9	0.4
CGA at discharge (wk)	36.6 ± 2.1	37.0 ± 2.5	37.1 ± 2.9	36.8 ± 1.9	2.3	0.1
Medical complications during hospital stay						
NRDS	150 (34.4)	133 (30.0)	129 (27.2)	131 (34.3)	17.1	0.1
BPD	56 (12.8)	56 (12.6)	85 (17.9)	48 (12.6)	15.1	0.2
NEC/sepsis	52 (11.9)	45 (10.2)	48 (10.1)	37 (9.7)	11.0	0.4
KMC initiation						
Age (d)	21 (11, 29)	21 (11, 28)	19 (12, 30)	18 (11, 29)	2.0	0.6
CGA (wk)	33.7 ± 2.1	33.5 ± 2.3	33.7 ± 2.2	33.8 ± 2.1	0.8	0.5
Weight (g)	1620.9 ± 300.6	1650.7 ± 612.5	1614.5 ± 334.7	1674.4 ± 515.7	1.4	0.2
Respiratory support (%)^a^	146 (34.4)	142 (30.1)	197 (41.0)	140 (32.6)	0.4	0.5
By mother (%)	389 (79.6)	428 (77.8)	429 (77.0)	399 (80.3)	2.1	0.5
KMC sessions	8.8 (5, 12)	9.4 (5, 11)	7.0 (5, 11)	6.0 (5, 9)	8.4	< 0.05
Average cumulative hours of KMC received per infant (h)	12 (5.3, 28.1)	12 (5.7, 21.3)	14 (9.0, 28.8)	14 (8.0, 22.9)	13.9	< 0.05
Pause KMC due to unstable vital signs (%)	1.0 ± 0.1	0.9 ± 0.3	0.5 ± 0.2	0.8 ± 0.4	17.2	0.1

Data are mean ± standard deviation, median (first quartile, third quartile) and *n* (%). *CGA* corrected gestational age, *NRDS* neonatal respiratory distress syndrome, *BPD* bronchopulmonary dysplasia, *NEC* necrotizing enterocolitis in neonates, *KMC* kangaroo mother care. ^a^The percentage of preterm infants that require respiratory support at their first KMC session including nasal or non-invasive ventilation

On average, there were 33 beds in each NICU (Supplementary Table 2). The average number of KMC lounge chairs per NICU increased from 7.5 in March 2018 to 10.6 in March 2019. In terms of human resources, the average number of nurses that received KMC training per NICU increased from 61.9 to 67.1, while the number of nurses that could support provision of KMC per NICU increased from 23.1 to 43.3, with a statistically significant difference (*P* < 0.05). Healthcare workers assisted parents during the KMC process (53.7% adjusting feeding posture, 80.2% putting baby on chest and 75.7% o﻿bserving baby signs) (Supplementary Fig. 3). Support from family member ranged from 93.4% to 99.3%, with a mild rise in the end, but the increase was not statistically significant. The proportion of willing to recommend KMC for the others was between 91.3% and 97.1% (Supplementary Table 3).

The percentage of successful follow-up varied between 89.8% and 96.5% during the 12-month period with a slightly increasing trend (Supplementary Table 4, *P* < 0.05). The percentage of infants who continued to receive KMC post-discharge increased from 33.7% in April 2018 to 65.8% in March 2019 (*P* < 0.05). The effects of different measures on infant’s weight gain of KMC post-discharge are shown in Table 2. There are four predictors had a significant effect on growth weight: gender, GA, feeding and whether gained severe illness during hospital (*P* < 0.05). Among these, male infants showed higher growth rate than female infants. Given that the overall analysis of infants might partially offset the GA difference, no effect was observed in those who continued to receive KMC post-discharge. We focused subsequent analyses on younger GA. Most KMC infants have a 50-percentile birth weight, though the weight at discharge fell to 10-percentile of the Fenton growth chart (Fig. 1 and Supplementary Table 5). The follow-up weights of infants who continued to receive KMC post-discharge were higher than those who did not continue to receive KMC post-discharge for infants born at 28–29 weeks (3246.4 vs. 2706.2 g, *P* < 0.05); however, this trend was not significant in larger GA preterm infants (Fig. 1).

**Table 2:** The effect of predictors on growth weigh of infants after discharge

Variables	Main effect		Time		Interactive effect		Statistical value	Rate (95% CI)
	Beta (95% CI)	*P*	Beta (95% CI)	*P*	Beta (95% CI)	*P*		
Gender	−101.03 (−187.30, −14.75)	0.02	10.22 (9.22, 11.22)	< 0.01	4.97 (3.50, 6.44)	< 0.01	1 = boy	15.19 (12.72, 17.65)
							2 = girl	10.22 (12.63, 14.45)
Gestational age class	102.06 (9.06, 195.07)	0.03	13.54 (12.63, 14.45)	< 0.01	5.27 (3.33, 7.21)	< 0.01	1 = 32– < 37 wk	18.81 (15.96, 21.67)
							2 = < 32 wk	13.54 (12.63, 14.45)
NRDS	68.52 (−23.95, 160.99)	0.15	13.48 (12.48, 14.47)	< 0.01	−2.72 (−4.24, −1.21)	< 0.01	1 = yes	10.76 (8.25, 13.26)
							2 = no	13.48 (12.48, 14.47)
Apnea	24.32 (−147.16, 195.80)	0.78	12.52 (11.73, 13.32)	< 0.01	−3.38 (−6.01, −0.75)	0.01	1 = yes	9.15 (5.72, 12.57)
							2 = no	12.52 (11.73, 13.32)
BPD	−554.25 (−763.10, −345.40)	< 0.01	12.43 (11.60, 13.25)	< 0.01	4.89 (2.15, 7.63)	< 0.01	1 = yes	17.32 (13.75, 20.89)
							2 = no	12.43 (11.60, 13.25)
NEC/sepsis	446.39 (318.36, 574.43)	< 0.01	16.12 (15.25, 17.00)	< 0.01	−10.16 (−11.88, −8.43)	< 0.01	1 = yes	5.97 (3.38, 8.56)
							2 = no	16.12 (15.25, 17.00)
Feeding	94.85 (7.48, 182.23)	0.03	13.29 (12.20, 14.38)	< 0.01	−2.11 (−3.60, −0.62)	< 0.01	1 = exclusive breast	11.18 (8.60, 13.77)
							2 = mixed	13.29 (12.19, 14.40)
Whether continue KMC	−44.36 (−132.04, 43.32)	0.32	12.10 (11.02, 13.18)	< 0.01	−0.02 (−1.51, 1.46)	0.98	1 = yes	12.08 (9.51, 14.65)
							2 = no	12.10 (11.01, 13.18)

*NRDS* neonatal respiratory distress syndrome, *BPD* bronchopulmonary dysplasia, *NEC* necrotizing enterocolitis in neonates, *KMC* kangaroo mother care, *CI* confidence interval

**Fig. 1:**
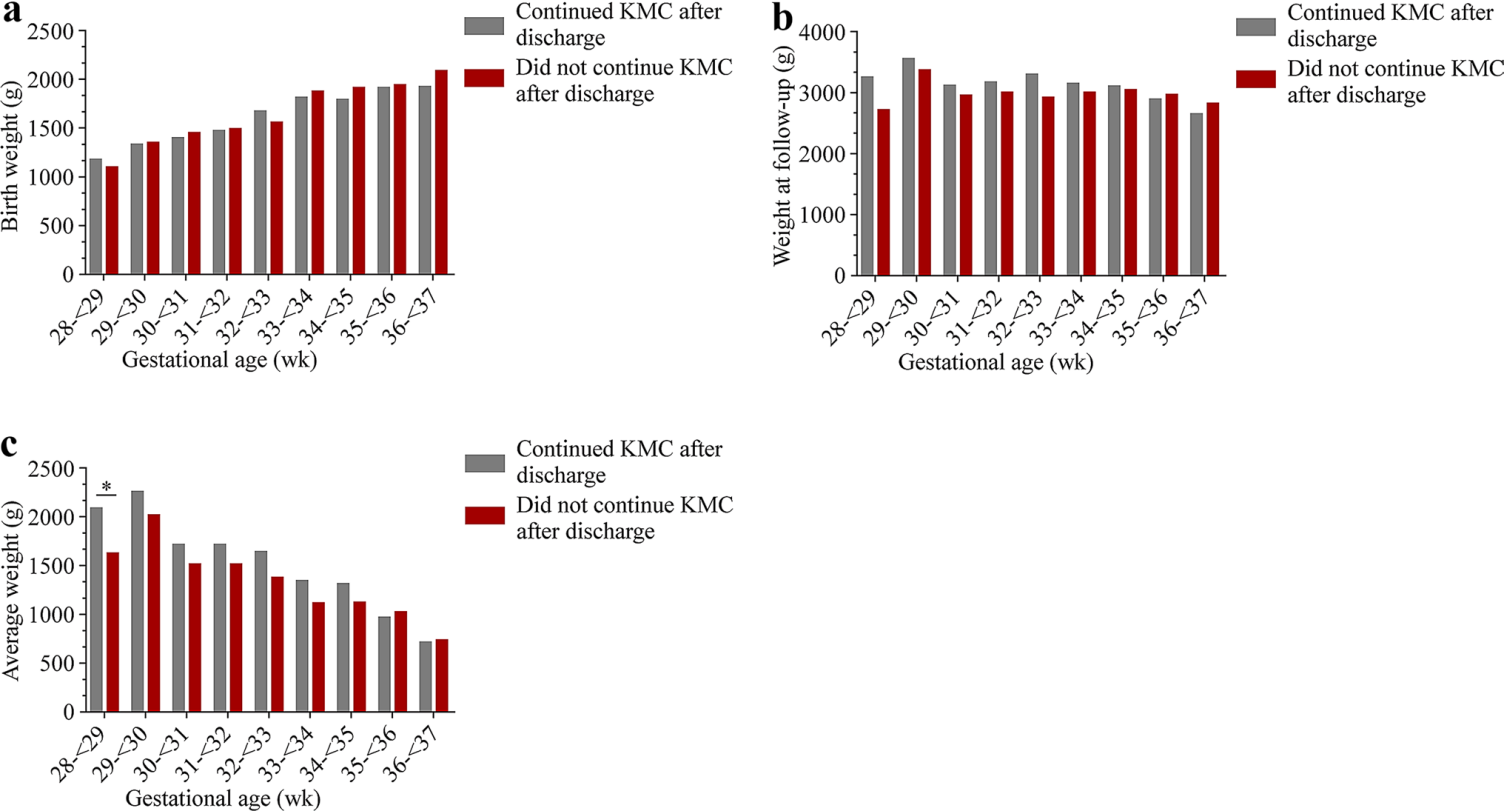
Body weight changes at follow-up visits. **a** Birth weight of KMC infants; **b** follow-up weight of KMC infants at gestational age 40–41 weeks; **c** the average weight gain of KMC infants. *KMC* kangaroo mother care

In this study, we reviewed facility capacity to provide KMC, the characteristics of preterm infants admitted to the NICUs, the proportion who received KMC, weight gain and patterns of KMC provision. Adequate spacing, equipment and human resources are key points to successful KMC implementation. Due to the practice of restricting parental access to NICUs in China, the KMC protocol developed was for intermittent KMC [8, 9]. Despite the improved service capacity, we did not observe an increase in KMC coverage, which is contrary to the perspective that hospital infrastructure is the main barrier to KMC implementation. Qualitative research from our implementation project suggested that other barriers at cultural, parental and financial levels might impede expanding coverage [10]. For example, many families in China still follow the cultural practice of “doing-the-month” where the new mothers must stay indoors; therefore, mothers who have been discharged home are hesitant to travel to the hospital to participate in KMC [11–13]. One potential solution to this cultural barrier is asking fathers to participate in KMC. Studies from Shorey et al. and Deng et al. suggested that KMC provided by fathers also could stabilize infants’ vital signs, reducing pain and relieving parental anxiety, and potentially could improve neurological prognosis of preterm infants [14–16]. Based on the current follow-up model of preterm infants in China, most infants who received KMC could be successfully followed up (96%). Most preterm infants had a CGA below 37 weeks when discharged; therefore, continued KMC is recommended [17]. The follow-up weight of preterm infants that continued KMC post-discharge was also significantly larger than those who did not continue KMC, especially in smaller GA groups. Previous studies also exhibited satisfactory weight gain of preterm infants who practiced KMC post-discharge [18, 19]; therefore, medical staff should continue educating parents of KMC continuum’s value on preterm outcomes and should encourage parents to continue practicing KMC post-discharge.

This study has the following limitations. We presented the aggregated data across eight NICUs but there could have been inconsistency among hospitals regarding KMC implementation and infant discharge policy. However, we believe that our aggregated data could provide the best-available information on KMC utilization in China’s NICUs.

In conclusion, we found around one-quarter of all preterm infants were receiving KMC, especially those with lower birth weight and lower GA at birth. The proportion of preterms that received KMC did not change during the 12-month period despite the increase in units’ service capacity. We recommend a more in-depth study of hospital context, process and additional resources required if KMC is to be scaled up further in China’s NICUs.

## Supplementary Information

Below is the link to the electronic supplementary material.

Supplementary file 1 (PDF 317 KB)

## Data Availability

All authors approve to deposit data that support the findings of their research in a public repository.
