# Supplementary material for: Implementation experience of a 12-month intervention to introduce intermittent kangaroo mother care to eight Chinese neonatal intensive care units
Source: World J Pediatr. 2022 Aug 25;18(12):849–53. doi: 10.1007/s12519-022-00607-4 (PMC9617830; doi:10.1007/s12519-022-00607-4)
Supplement: Supplementary file 1 — (PDF 317 KB) [file 12519_2022_607_MOESM1_ESM.pdf]

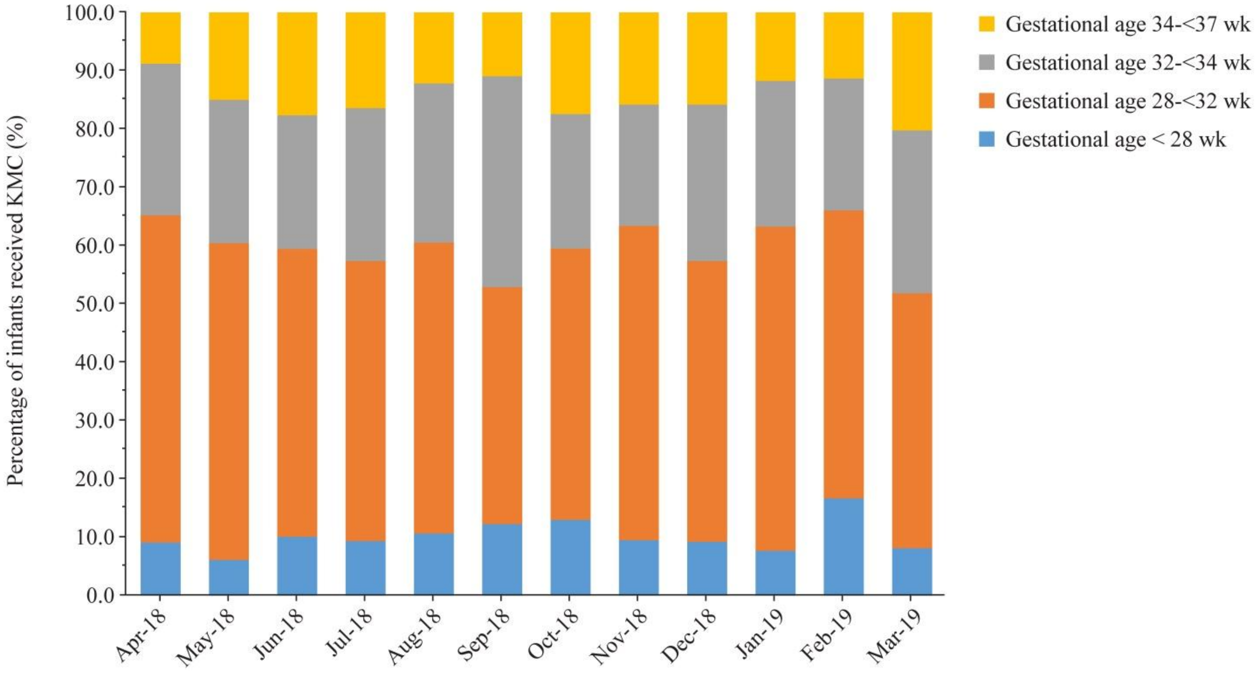

Supplementary Fig. 1 Percentage of infants who received KMC by gestational age at birth. *KMC* kangaroo mother care

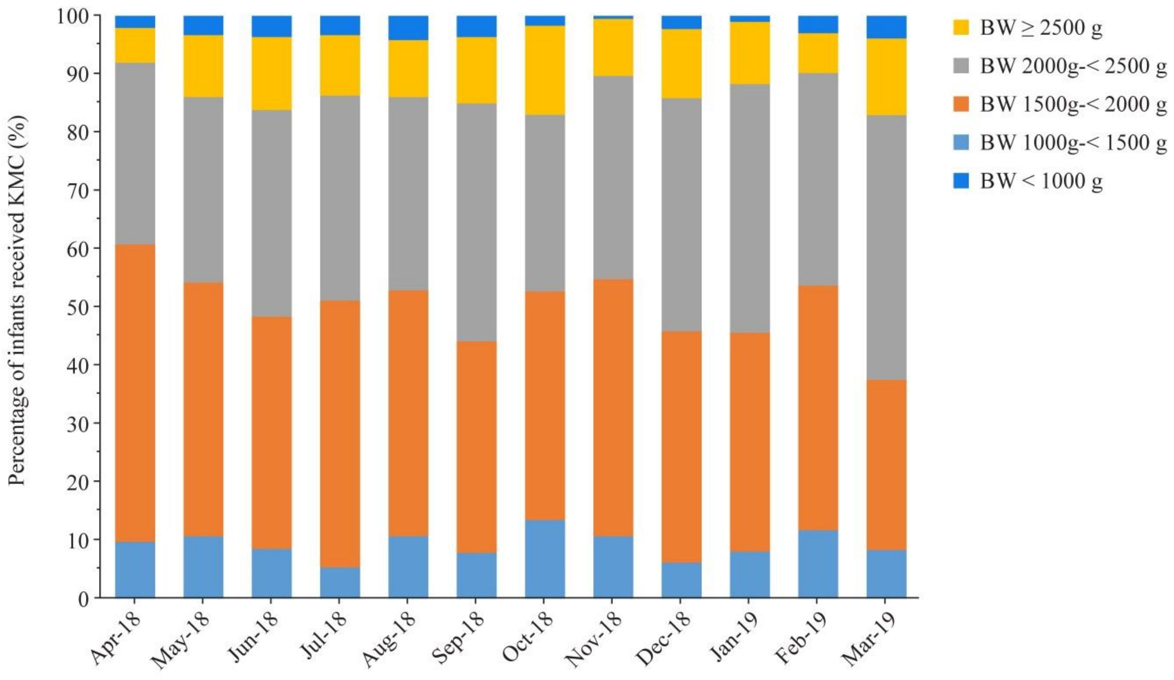

Supplementary Fig. 2 Percentage of infants who received KMC by birth weight. *KMC* kangaroo mother care, *BW* birth weight

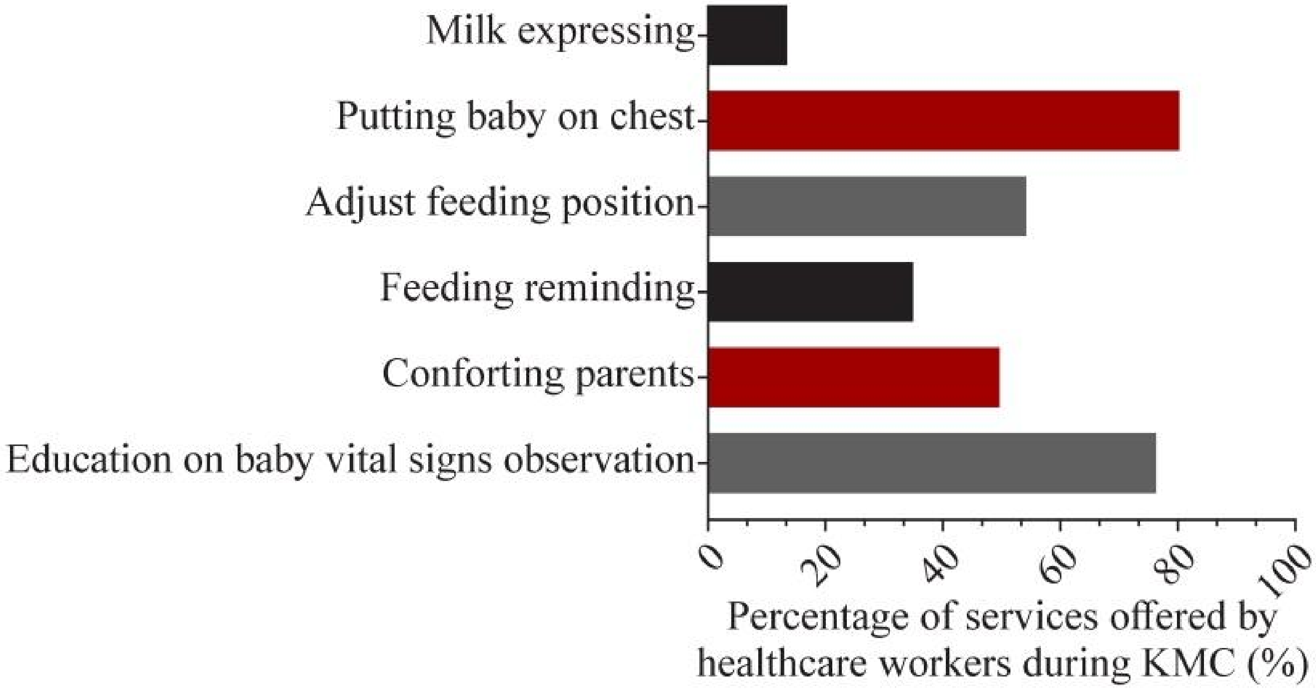

Supplementary Fig. 3 The percentage of different kinds of care process provided by medical staff. *KMC* kangaroo mother care

Supplementary Table 1. Total number of preterm infants admitted to the NICUs and the number who received KMC

Quarter		Total			Q1: Apr-18 to Jun-18			Q2: Jul-18 to Sep-18			Q3: Oct-18 to Dec-18			Q4: Jan-19 to Mar-19		
Items		Number	KMC number	%	Number	KMC number	%	Number	KMC number	%	Number	KMC number	%	Number	KMC Number	%
Total		8240	2093	25.4	2055	489	23.8	2120	550	25.9	2009	557	27.7	2056	497	24.2
Gestational age (wk)	< 32	2072	1239	59.8	499	299	69.0	536	312	58.2	525	332	63.2	512	296	57.8
	32-< 34	1771	543	30.7	434	119	27.4	446	164	36.7	430	133	30.9	461	127	27.5
	34-< 37	4397	311	7.1	1122	71	6.3	1138	74	6.5	1054	92	8.7	1083	74	6.8
χ^2				16,480			551.0			4240			522.4			496.6
P				< 0.05			< 0.05			< 0.05			< 0.05			< 0.05
Birth weight (g)	< 1500	1668	1036	62.1	440	263	59.8	426	270	63.4	425	282	66.4	377	221	58.7
	1500-< 2000	2267	769	33.9	528	161	30.5	607	201	33.1	541	198	36.6	591	209	35.4
	2000-< 2500	2587	228	8.8	650	49	7.5	656	58	8.8	655	68	10.4	626	53	8.5
	≥ 2500	1718	60	3.5	437	16	3.7	431	21	4.9	388	9	2.3	462	14	3.0
χ^2				24,720			519.6			526.5			561.0			481.4
P				< 0.05			< 0.05			< 0.05			< 0.05			< 0.05

NICU neonatal intensive care unit, *KMC* kangaroo mother care. % KMC premature/preterm infant discharged; χ^2 Chi-squared test

Supplementary Table 2. Service capacity in the first and last month of the study

Indicators		2018-03	2019-03	t/χ^2	P
Facility	NICU bed per NICU	33 (22, 60)	33 (22, 60)	NA	NA
	Bed utilization rate	112% ± 22.5%	117% ± 18.8%	-1.45	0.19
	KMC chair per NICU	7.5 (3, 15)	10.6 (6, 20)	-3.3	< 0.05
	Use of KMC recording sheet (%)[a]	2 (25)	6 (75)	-	0.13
Human recourse	Nurses that received KMC training per NICU	61.9 (30, 85)	67.1 (30, 91)	-0.9	0.41
	Nurses that could perform KMC per NICU	23.1 (7, 45)	43.3 (14, 60)	-2.6	0.04

Data are median (first quartile, third quartile), mean ± standard deviation or *n* (%). *NICU* neonatal intensive care unit, *KMC* kangaroo mother care, *NA* not available. [a] The number of hospitals that use the KMC recording sheet/8 (the total number of hospitals). *t* t test, χ^2 Chi-squared test

Supplementary Table 3. Parental recognition about KMC and nursing services

Items	Apr-18	May-18	Jun-18	Jul-18	Aug-18	Sep-18	Oct-18	Nov-18	Dec-18	Jan-19	Feb-19	Mar-19	χ^2	*P*
Get assistant from healthcare workers, %	99.2	96.8	99.4	97.0	98.5	98.6	98.0	98.8	98.6	96.5	96.1	98.6	10.1	0.5
Support from family members, %	93.4	94.2	96.3	95.3	97.0	99.3	97.3	97.6	98.7	97.9	97.1	99.3	19.0	0.1
Willingness to recommend KMC to others, %	91.7	92.9	93.8	91.1	96.3	93.5	96.6	92.9	94.3	93.6	91.3	97.1	10.7	0.5

KMC kangaroo mother care. χ^2 Chi-squared test

Supplementary Table 4. Post discharge follow-up of infants who received KMC

Month	Apr-18	May-18	Jun-18	Jul-18	Aug-18	Sep-18	Oct-18	Nov-18	Dec-18	Jan-19	Feb-19	Mar-19	χ^2/H	*P*
Percentage of successful follow-up, %	82.8	92.9	95.7[a]	96.5[a]	91.1	94.2[a]	95.2[a]	94.7[a]	90.1	90.8	95.1	94.1[a]	31.6	< 0.05
Percentage of continued KMC, %	33.7[b]	48.6	46.1	41.7[b]	39.8[b]	51.9	40.0[b]	33.8[b]	43.7[b]	51.6	55.1	65.8	45.7	< 0.05
KMC session per d	1 (1, 2)	1 (1, 2)	1(1, 2)	1 (1, 2)	1 (1, 2)	1 (1, 2)	1 (1, 2)	1 (1, 1.8)	1 (1, 1)	1 (1, 1)	1 (1, 1.8)	1 (1, 2)	-	-
KMC length per session (min)	50 (30, 60)	60 (30, 60)	60 (40, 60)	56 (30, 60)	30 (30, 60)	60 (30, 60)	60 (50, 60)	60 (30, 60)	50 (30, 60)	60 (30, 60)	60 (41.3, 60)	60 (30, 60)	-	-

KMC kangaroo mother care. χ^2 Chi-squared test. [a]Different when compared with Apr-18;[b]Different when compared with Mar-19.

Supplementary Table 5. Discharge weight of KMC infants

GA	Birth weight	Discharge GA	Discharge weight
28-<29	1175.9 ± 199.7	34-<35	1985 ± 160.6
		35-<36	2071.9 ± 223.1
		36-<37	2124.7 ± 245.2
		37-<38	2276.7 ± 325.1
		38-<39	2327.1 ± 353.1
		39-<40	2190.1 ± 397.9
29-<30	1297.3 ± 201.7	33-<34	1942 ± 110.9
		34-<35	2026.5 ± 198.0
		35-<36	2055.5 ± 281.4
		36-<37	2113.1 ± 269.4
		37-<38	2343.4 ± 685.4
		38-<39	2235.9 ± 290.5
30-<31	1427.6 ± 239.0	33-<34	1896.1 ± 198.4
		34-<35	1999.5 ± 160.1
		35-<36	2089.9 ± 195.4
		36-<37	2061.5 ± 257.8
		37-<38	2195.8 ± 350.7
		38-<39	2120.4 ± 272.7
		39-<40	2365.6 ± 312.1
31-<32	1568.5 ± 336.6	34-<35	2032.1 ± 212.4
		35-<36	2002.8 ± 195.5
		36-<37	2105.7 ± 280.2
		37-<38	2171.3 ± 335.2
		38-<39	2151.1 ± 306.9
32-<33	1636.1 ± 287.6	34-<35	2043.8 ± 225.2

		35-<36	2062.5 ± 196.1
		36-<37	2068.8 ± 265.7
		37-<38	2153.9 ± 240.5
		38-<39	2123.8 ± 201.9
		39-<40	2111.4 ± 341.1
33-<34	1788.9 ± 350.4	35-<36	2074.3 ± 214.7
		36-<37	2070.9 ± 296.2
		37-<38	2110.6 ± 251.4
		38-<39	1938.2 ± 230.3
34-<35	2006.3 ± 723.8	35-<36	2229.5 ± 242.5
		36-<37	2207.3 ± 331.7
		37-<38	2163.0 ± 358.3
		38-<39	2083.1 ± 346.6
35-<36	2101.5 ± 481.0	36-<37	2466.9 ± 465.4
		37-<38	2186.4 ± 308.7
		38-<39	2299.3 ± 605.7
36-<37	2157.8 ± 595.2	37-<38	2492.1 ± 416.5
		38-<39	2509 ± 416.2
		39-<40	2058.3 ± 341.0

Vales are mean ± standard deviation. *KMC* kangaroo mother care, *GA* gestational age
